# Mental health symptoms of youth initiating psychiatric care at different phases of the COVID-19 pandemic

**DOI:** 10.1186/s13034-022-00511-9

**Published:** 2022-09-30

**Authors:** Brent R. Crandal, Andrea L. Hazen, Kelsey S. Dickson, Chia-Yu Kathryn Tsai, Emily Velazquez Trask, Gregory A. Aarons

**Affiliations:** 1 Department of Psychiatry, University of California, 9500 Gilman Drive, La Jolla, San Diego, CA 92093 USA; 2 Behavioral Health Services, Rady Children’s Hospital, 3020 Children’s Way, San Diego, CA 92123 USA; 3 Chadwick Center for Children & Families, Rady Children’s Hospital, 3020 Children’s Way, San Diego, CA 92123 USA; 4 Child and Adolescent Services Research Center, 3665 Kearny Villa Road, Suite 200N, San Diego, CA 92123 USA; 5 Department of Child and Family Development, San Diego State University, 5500 Campanile Dr, San Diego, CA 92182 USA; 6 California University of Science and Medicine, 1501 Violet Street, Colton, CA 92324 USA; 7 Behavioral Health Services Department, Health and Human Services, County of San Diego, 3255 Camino del Rio South, San Diego, CA 92108 USA

## Abstract

**Objective:**

To examine differences in caregiver and youth reported mental health symptoms for youth initiating mental health treatment through phases of the Coronavirus Disease (COVID-19) pandemic, compared with symptomology reported the prior year.

**Study design:**

This retrospective study analyzes group differences in mental health symptoms (Pediatric Symptom Checklist; PSC-35) based on 7874 youth seeking treatment in publicly funded mental health treatment programs during California’s Stay-At-Home order (March–May, 2020) and the prolonged pandemic (May–December, 2020) phases of the COVID-19 pandemic as compared with matching groups in 2019.

**Results:**

Youth entering mental health treatment services, and their caregivers, reported significantly increased internalizing, externalizing, and attention-related symptoms during the prolonged pandemic phase, but not during the acute stay-at-home phase of the COVID-19 pandemic, and with small effect sizes. Group comparison analyses did not detect a significantly larger effect for Sexual and Gender Diverse (SGD) youth who identify as lesbian, gay, bisexual, asexual, transgender, Two-Spirit, queer, and/or intersex, and Black, Indigenous, People of Color (BIPOC).

**Conclusions:**

A large-scale comparison of youth mental health symptoms before and during the COVID-19 pandemic suggests that mental health was disrupted for youth seeking treatment as the pandemic prolonged throughout 2020.

## Introduction

In the United States, the first coronavirus disease 2019 (COVID-19) patient was identified in late January 2020. By June 2020, the US was leading the world with the highest number of both COVID-19 disease incidence and related deaths [1, 2]. Due to California’s alarming disease incidence, individual counties eventually placed “stay-at-home” (SAH) orders on its constituents and began suggesting or requiring protective face masks to be worn in public spaces. Accompanying these public health interventions, California school districts and non-essential businesses closed in adherence to both federal and state executive orders requiring everyone to stay within their residences to reduce COVID-19 spread.

A notable time period encompassing these major changes began the week of March 16, 2020 until May 22, 2020 when California state governor issued a SAH order across the state in order to slow the spread of COVID-19 [3]. Despite disease prevention measures, COVID-19 disease incidence continued to surge across the nation, leading to an abrupt reduction in adult workforce, dramatic changes to educational systems, and a continuous increase in COVID-19 disease incidence in healthcare institutions. In response to the continued increase of COVID-19, many countries, states, or regions re-issued disease control methods. On November 21, 2020 California issued a limited SAH order which was in effective until December 21, 2020, accompanied by a regional stay-at-home order on December 5 to keep COVID-19 spread from overwhelming hospital intensive care unit capacity [4].

### COVID-19 and youth mental health

While the immediate impact of the pandemic on the mental health (MH) of youth rippled throughout communities and families, there were large gaps in knowledge about the MH impacts of the pandemic on children and adolescents [5]. Standardized reviews have shown COVID-19 was related to greater depression, anxiety, and behavioral problems among young people [6–10]. The impact of pandemic-caused proximal risk factors (e.g., worry about imminent risk of COVID-19 infection, fear for the safety of loved ones) and distal risk factors (e.g., financial, housing and food uncertainty, heightened and prolonged burden on parents, less access to supportive and social services, school or extracurricular program closure, social isolation) on youth well-being were of significant concern [11–16]. This was further underscored by findings from Palinkas et al. [17] highlighting the unmet MH service needs associated with significantly reduced out-of-home services, such as outpatient MH care in response to COVID-19, especially in states with high COVID-19 incidence rates.

During the pandemic, researchers also reported a rise in MH-related emergency department (ED) visits [18], as well as increases in suicidal ideation and attempts among youth seen in pediatric emergency departments [19, 20]. The increase in MH-related ED visits was striking given the general decrease in non-COVID related ED visits during the same time period [21]. Preliminary findings also suggest a prolonged impact of the pandemic, with the potential for an enduring impact on children’s MH symptoms (e.g., Post-Traumatic stress, depressive, and anxiety disorders, as well as grief-related symptoms [22–24]). Limited emerging research with adults supports the prolonged MH impact of COVID-19 among adults, especially among those with fewer economic resources and/or COVID-19 related stressful life events (e.g., job loss, death of a family member [25, 26]). As a result, there is a significant need and urgent calls for efforts further evaluating and addressing the impact, especially long-term impact, of COVID-19 on youth’s MH [27].

### COVID-19, mental health and diverse youth

Some have predicted that quarantine measures are likely to amplify symptoms for youth with existing psychiatric conditions, extending beyond the pandemic [22, 28, 29]. Many have also called for greater support and consideration of diverse youth (e.g., Sexual and Gender Diverse [SGD] youth who identify as lesbian, gay, bisexual, asexual, transgender, Two-Spirit, queer, and/or intersex, and Black, Indigenous, People of Color [BIPOC]) in the COVID-19 public health response [28, 30, 31]. The sparse COVID-19-related literature with SGD youth reports increased MH challenges related to SAH or social distancing orders [32, 33], while the findings related to BIPOC youth are mixed. Some findings are suggestive of greater impact on BIPOC youth while others suggest limited changes or even improved MH symptoms [34, 35]. Literature examining psychiatric impacts of COVID-19 on SDG and BIPOC adults shows higher levels of depression, trauma-related symptoms, and greater increases in general MH symptoms [36–38]. Additional research highlights the disproportionate impact on adults with existing psychiatric conditions, especially among those who are SGD [36, 39]. The paucity of literature with youth, however, bares a critical gap in our understanding of the MH impact of the COVID-19 pandemic on SGD and BIPOC youth who are at risk for discrimination and who encounter disproportionate barriers to appropriate psychiatric care.

**Fig. 1:**
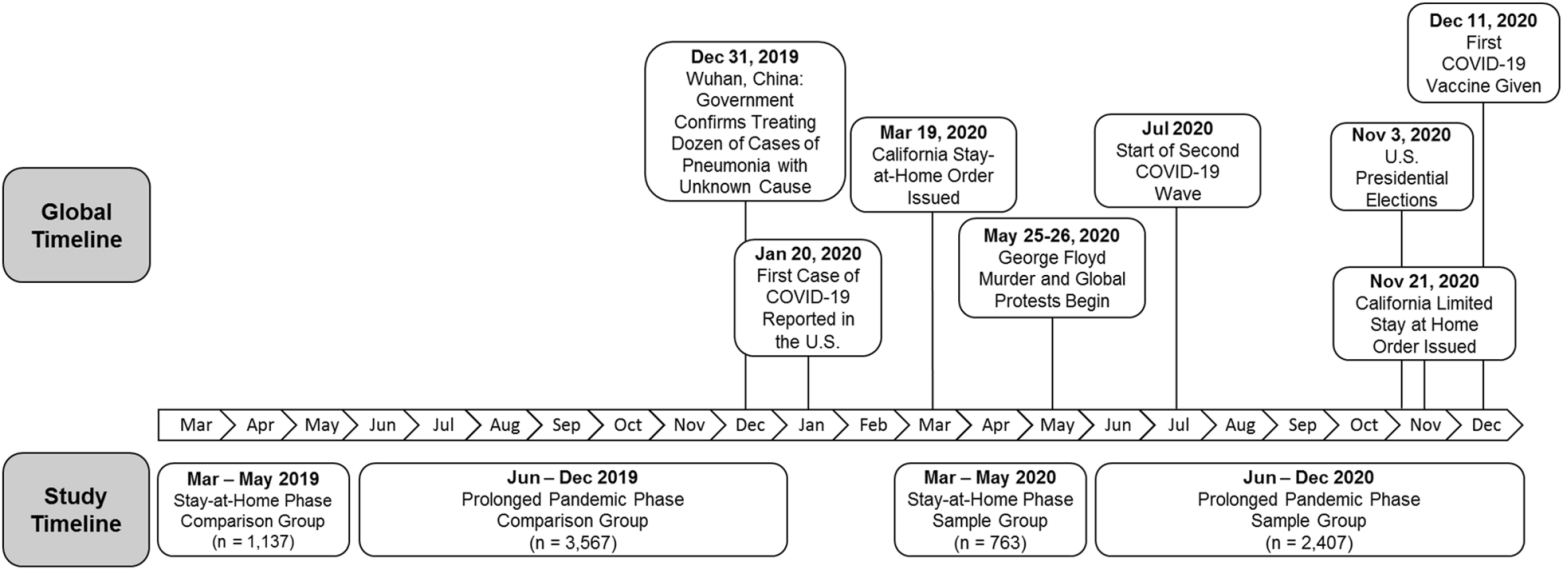
COVID-19 pandemic and study timelines

In response to the critical need to understand and act on the immediate and prolonged MH impact on youth, we examined differences in MH symptom reporting during the approximately 2-month California SAH order in 2020 and the subsequent 7 months following the SAH order compared with an identical timeframe in 2019 (see Fig. 1). In this study we hypothesize: (a) both caregiver and youth report of MH symptom acuity at intake for youth seeking outpatient MH services would be higher during the COVID-19 pandemic SAH order compared to the same time frame the previous year; (b) caregiver and youth report of MH symptom acuity at intake for youth seeking outpatient MH services would be higher in the prolonged months of the COVID-19 pandemic (after SAH order until the end of the 2020 calendar year) compared to the same time frame the previous year; (c) caregiver and youth MH symptom acuity at intake for youth seeking outpatient MH services during both phases of the pandemic will be disproportionately higher for SGD or BIPOC youth, compared to the same time frame the previous year.

## Methods

### Study context and procedure

Data were collected from youth who received publicly funded outpatient MH services and their parent/caregiver in the 5th largest county in the United States. Administrative data used in this study were collected by the county as part of ongoing evaluation activities. The sample includes youth ages 6 to 17 years of age who initiated services and had an intake Pediatric Symptom Checklist (PSC-35) completed by a caregiver during the 3 months of the SAH in California, in the subsequent 7 months following the SAH order (Prolonged Pandemic (PP)), or in comparable time periods in 2019 (see Fig. 1). For youth ages 11 to 17 years, data from a self-reported intake PSC-35 were included in the analyses if the youth measure was completed within the same time period in which the caregiver measure was completed. The time periods used in the study are: (a) between March 16, 2020 and May 22, 2020 (SAH cohort); (b) between March 16, 2019 and May 22, 2019 (SAH comparison cohort); (c) between May 23, 2020 and December 31, 2020 (PP cohort); and (d) between May 23, 2019 and December 31, 2019 (PP comparison cohort). The sample contains unique cases as youth were unduplicated both within and across all time periods. Gender, race, ethnicity, and sexual orientation data are routinely collected in clinical settings by MH providers at initial diagnostic evaluations and recorded in the electronic health record via a standardized initial diagnostic evaluation tool [40]. There are required trainings and quality assurance checks used at the County-level to promote accuracy of the data for clinical and administrative uses [41].

Fifty-four county contracted programs provided services to youth in either the 2020 SAH cohort (n = 45 programs, 83.3%) or 2019 pre-pandemic SAH comparison cohort (n = 50 programs, 92.6%). Forty-one of the programs (75.9%) provided services in both the SAH and comparison time periods. Fifty-two programs provided services to youth in either the 2020 PP cohort (n = 48 programs, 92.3%) or the 2019 pre-pandemic PP comparison cohort (n = 52 programs, 100.0%). Forty-eight of the programs (92.3%) provided services in both the PP and comparison time periods. Measures were administered by either clinicians or administrative staff at each clinical site and then data were entered in a Health Insurance and Portability and.

Accountability Act-compliant web-based data entry system. Administration guidelines specify that intake measures are to be completed and data entered within 30 days of the initial service date. Study data were extracted from the county’s MH system databases in August 2021. The study was approved by the Institutional Review Board at University of California, San Diego.

### Sample

The sample included 7874 youth distributed as follows: 763 youth in the 2020 SAH cohort; 1137 youth in the 2019 pre-pandemic SAH comparison cohort; 2407 youth in the 2020 PP cohort; and 3567 youth in the 2019 pre-pandemic PP comparison cohort. Demographic information for the SAH cohort and comparison cohort is provided in Table 1, and information for the PP cohort and comparison cohort is provided in Table 2. Of the 7874 participants, 4981 youth ages 11–17 had data from the PSC-35 youth self-report as follows: 998 in the 2020 SAH cohort; 699 in the 2019 SAH comparison cohort; 1595 in the 2020 PP cohort; and 2188 in the 2019 PP comparison cohort.

**Table 1:** Stay-at-home sample demographics and tests for group differences

	Total sample (*n* = 1900)	2019 SAH cohort (*n* = 1137)	2020 SAH cohort (*n* = 763)	Group differences
	*n* (%)	*n* (%)	*n* (%)	*p*
**Youth age (years)**				n.s
6–10	645 (34)	397 (35)	248 (33)	
11–13	557 (29)	344 (30)	213 (28)	
14–17	698 (37)	396 (35)	302 (40)	
**Youth gender**				n.s
Female	910 (48)	547 (48)	363 (48)	
Male	915 (48)	551 (48)	364 (48)	
Non-binary, trans, queer, other	55 (3)	31 (3)	24 (3)	
**Youth race/ethnicity**				n.s
Black	89 (5)	57 (5)	32 (4)	
Hispanic	1251 (66)	736 (65)	515 (67)	
Multiracial	152 (8)	96 (8)	56 (7)	
Other	98 (5)	66 (6)	32 (4)	
White	299 (16)	176 (15)	123 (16)	
**Youth sexual orientation**				n.s
LGBTQ	275 (14)	164 (14)	111 (15)	
Heterosexual/straight	1272 (67)	758 (67)	514 (67)	
**Youth relationship to caregiver**				n.s
Adoptive parent	86 (5)	50 (4)	36 (5)	
Biological parent	1556 (82)	928 (82)	629 (82)	
Foster parent	86 (5)	51 (4)	35 (5)	
Other	153 (8)	93 (8)	60 (8)	

A continuous age variable was used for the group comparison test. There were 20 (1.1%) cases with unknown/not reported gender (2019 Cohort *n* = 8; 2020 Cohort *n* = 12). There were 11 (0.6%) cases with unknown/not reported Youth Race/Ethnicity (2019 Cohort *n* = 6; 2020 Cohort *n* = 5). Due to small number of cases, Youth Race/Ethnicity “Other” category also includes responses of Asian/Pacific Islander and Native American. There were 353 (19%) cases of unknown/not reported Sexual Orientation (2019 Cohort *n* = 215; 2020 Cohort *n* = 138). LGBTQ = Lesbian, Gay, Bisexual, Trans, Queer & Questioning. There were 19 (1.0%) cases with unknown/not reported Caregiver Relationship to Youth (2019 Cohort *n* = 15; 2020 Cohort *n* = 3). Due to small number of cases, Caregiver Relationship to Youth “Other” category also includes responses of Other Family Member (non-foster status), Staff (Residential Programs)

**Table 2:** Prolonged pandemic sample demographics and tests for group differences

	Total sample (*n* = 5974)	2019 prolonged cohort (*n* = 3567)	2020 prolonged cohort (*n* = 2407)	Group differences
	n (%)	*n* (%)	*n* (%)	*p*
**Youth age (years)**				.002
6–10	2018 (34)	1278 (36)	740 (31)	
11–13	1755 (29)	1034 (29)	721 (30)	
14–17	2201(37)	1255 (35)	946 (39)	
**Youth gender**				< .001
Female	2850 (48)	1591 (45)	1259 (52)	
Male	2834 (47)	1797 (50)	1037 (43)	
Non-binary, trans, queer, other	206 (3)	119 (3)	87 (4)	
**Youth race/ethnicity**				n.s
Black	340 (6)	210 (6)	130 (5)	
Hispanic	3947 (66)	2379 (67)	1568 (65)	
Multiracial	401 (7)	230 (6)	171 (7)	
Other	241 (4)	154 (4)	87 (4)	
White	1009 (17)	570 (16)	439 (18)	
**Youth sexual orientation**				< .001
LGBTQ	977 (16)	518 (15)	459 (19)	
Heterosexual/straight	3826 (64)	2349 (66)	1477 (61)	
**Youth relationship to caregiver**				n.s
Adoptive parent	304 (5)	178 (5)	126 (5)	
Biological parent	4935 (83)	2951 (83)	1984 (82)	
Foster parent	233 (4)	127 (4)	106 (4)	
Other	427 (7)	257 (7)	170 (7)	

A continuous age variable was used for the group comparison test. There were 84 (1.4%) cases with unknown/not reported gender (2019 Cohort *n* = 60; 2020 Cohort *n* = 24). There were 36 (0.6%) cases with unknown/not reported Youth Race/Ethnicity (2019 Cohort *n* = 24; 2020 Cohort *n* = 12). Youth race/ethnicity “Other” category also includes responses of Asian/Pacific islander and Native American due to small number of participants. There were 1171 (19.6%) cases of unknown/not reported sexual orientation (2019 cohort *n* = 700; 2020 cohort *n* = 471). LGBTQ = Lesbian, Gay, Bisexual, Trans, Queer & Questioning. There were 75 (1.3%) cases with unknown/not reported caregiver relationship to youth (2019 Cohort *n* = 54; 2020 Cohort *n* = 21). Due to small number of cases, caregiver relationship to youth “Other” category also includes responses of other family member (non-foster status), staff (residential programs)

The PSC-35 was completed in English by 74.8% of caregivers, 24.6% completed the measure in Spanish, and less than 1% completed in other languages (i.e., Arabic, Farsi, Tagalog, and Vietnamese). The PSC-35 youth self-report was completed in English by 96.7% of eligible youth, 3.2% completed it in Spanish, and 0.1% completed in other languages (i.e., Arabic, Tagalog, and Vietnamese).

### Measurement

Pediatric Symptom Checklist (PSC-35). The PSC-35 [42] was initially developed as a screening tool to help pediatric primary care providers identify youth who may be experiencing psychosocial problems and could benefit from further assessment. Through further psychometric evaluation, the utility of the tool has expanded to include outcome measurement and changes in symptoms over the course of treatment in outpatient pediatric psychiatry settings [43, 44]. The PSC-35 was selected for statewide use in California’s publicly-funded outpatient MH programs after an environmental scan of existing tools, provider surveys, literature review, a modified Delphi panel, and a systematic rating process for each measurement option [45], and was subsequently implemented in the county’s publicly-funded outpatient MH programs in July 2018 to continuously measure clinically significant levels of MH symptomology among child and youth participating in services. Psychometric performance of the PSC-35 has been well established in a variety of contexts, including pediatric and publicly-funded MH care settings and with diverse populations [46–52]. Researchers have found consistently higher scores reported for youth living in poverty, living in single-parent homes, or with parents who are mental ill [53].

In this study, the parent/caregiver version of the PSC-35 was administered to all caregivers of youth ages 6 to 17 years, and the youth self-report version was administered to youth ages 11 to 17 years. The tool consists of 35 items rated on a 3-point Likert scale (i.e., 0 = Never; 1 = Sometimes; 2 = Often) providing a total score from 0 to 70 as well as 3 subscale scores related to primary dimensions of MH symptomology: attention, internalizing (anxiety/depression) and externalizing (conduct) problems. Average scores from three published studies with participants from outpatient pediatric psychiatry treatment settings reported intake caregiver scores ranged from 25.7 to 27.4 [46, 52, 53], with clinical cutoff scores at intake in a treatment-seeking sample of youth (total score  ≥ 28; attention score  ≥ 7; internalizing  ≥ 5; externalizing score  ≥ 7). A change of  ± 6 points or more in the total score or of  ± 2 points or more in any subscale is psychometrically reliable change (i.e., signaling real change in symptoms) [52].

### Statistical analysis

Analyses were conducted using Stata statistical software, version 16.1 [54]. Differences between each pandemic cohort and its corresponding comparison cohort on youth age, gender, race/ethnicity, sexual orientation, and relationship to caregiver were evaluated using chi-square tests. Analysis of variance (ANOVA) or analysis of covariance (ANCOVA) was performed to examine differences between pandemic and pre-pandemic cohorts on the PSC total score, and multivariate analysis of variance (MANOVA) or multivariate analysis of covariance (MANCOVA) was used to examine differences on the three PSC subscale scores. When MANCOVA results suggested significant differences among PSC subscales, an ANCOVA was run for each subscale. Predictive margins were used to examine interactions between time cohort and youth gender, race/ethnicity, and sexual orientation while controlling for covariates [55]. Separate analyses were conducted for caregiver reported and youth reported PSC scores.

## Results

### Stay-at-home (SAH) results

There were no significant differences identified between the 2020 SAH cohort and 2019 SAH comparison cohort on youth age, gender, race/ethnicity, sexual orientation, or relationship to caregiver (see Table 1). Group mean comparison analyses showed no significant differences between the 2020 SAH cohort and 2019 comparison cohort on caregiver- and youth-reported PSC total or subscale scores. This suggests there were no increases in initial MH symptoms for those entering care during the SAH order period. Subsequent interaction effect analyses also showed no time effect (*p* > 0.05*)* on caregiver and youth reported PSC total or subscale scores based on an interaction with youth gender, race/ethnicity, or sexual orientation, respectively. In other words, MH symptom acuity was also not significantly higher for SGD and BIPOC youth who entered care during the SAH order period.

### Prolonged pandemic (PP) results

Preliminary analyses identified significant differences between the 2020 PP cohort and 2019 PP comparison cohort on youth age, gender, and sexual orientation. As a result, analyses for the PP sample (ANCOVA/MANCOVA) included these variables as covariates. Table 3 provides means, standard deviations, ANCOVA F Value Probabilities (*p*), and Effect Sizes (Eta-squared) for all PSC scale scores based on time cohort. ANCOVA results showed statistically significant differences between time points for PSC total scores rated by youth [*F*(13,315) = 25.38, *p* < 0.001] and caregivers [*F*(14,619) = 8.65, *p* = 0.003] while controlling for youth age, gender, and sexual orientation. MANCOVA results suggested significant increases from 2019 to 2020 PP cohorts for youth [*F*(33,315) = 18.20, *p* < 0.001] and caregiver [*F*(34,619) = 29.53, *p* < 0.001] ratings of attention and internalizing symptomology while controlling for the covariates.

**Table 3:** Prolonged pandemic sample means, standard deviations, ANCOVA F value probabilities and effect sizes for pediatric symptom checklist (PSC-35) scores based on time cohort

	2019	2020	ANCOVA F value	Effect size
	M ± SD	M ± SD	*p*	Eta-squared
Youth PSC-35				
Total	25.8 ± .23	27.9 ± .26	< .001	.01
Attention	4.7 ± .05	5.2 ± .06	< .001	.01
Internalizing	4.5 ± .06	5.2 ± .07	< .001	.01
Externalizing	3.1 ± .06	3.1 ± .06	n.s	–
Caregiver PSC-35				
Total	26.0 ± .20	27.0 ± .23	.003	< .01
Attention	4.3 ± .04	4.6 ± .06	< .001	.01
Internalizing	4.3 ± .04	4.8 ± .06	< .001	.01
Externalizing	4.8 ± .06	4.4 ± .07	.024	< .01

Subsequent ANCOVA analyses showed youth from the 2020 PP cohort reported significantly higher attention scores [*F*(13,315) = 36.98, *p* < 0.001] and internalizing scores [*F*(1335) = 39.34, *p* < 0.001] in 2020 compared with the same time period in 2019 while controlling for the covariates. Similarly, caregiver-rated PSC attention scores [*F*(14,619) = 31.18, *p* < 0.001] and internalizing scores [*F*(14,619) = 37.29, *p* < 0.001] were significantly higher in the 2020 PP cohort than in 2019 PP cohort while controlling for youth age, gender, and sexual orientation. On the other hand, caregivers reported reduced externalizing symptoms [*F*(14,619) = 5.09, *p* = 0.024] during the prolonged phase of the pandemic 2020, compared with 2019 while controlling for the covariates. Across significant ANCOVA results, all effect sizes were small as Eta-squared values were consistently at 0.01, except for caregiver-reported total score and externalizing score differences with Eta-squared values less than 0.01.

Using ANCOVA and MANCOVA tests, we also explored the effects of time period interacting with youth gender, race/ethnicity, or sexual orientation. There were no significant caregiver or youth-rated score interaction effects for gender or sexual orientation, or for youth report and race/ethnicity, but there was a significant interaction between time period and race/ethnicity for caregiver-reported total scores [*F*(4,4596) = 3.34, *p* = 0.010, Eta-squared < 0.01] and caregiver-reported internalizing scores [*F*(4,4596) = 3.28, *p* = 0.011, Eta-squared = 0.01]. With post-hoc marginal effects analyses, the “Other” Youth Race/Ethnicity [*F*(1,4583) = 9.53, *p* = 0.002], category was associated with statistically significant higher PSC total scores, but not internalizing scores, in 2020 compared with 2019 while controlling for youth age, and sexual orientation. The average marginal effect of time period on PSC total score is 6.903 points higher when Youth Race/Ethnicity was “Other.” The Other category was comprised of youth who reported other, native American, or Asian and Pacific islander as their race/ethnicity. The scores for Asian American and Pacific islander youth could be driving this finding, given the broader Asian American and Pacific islander community increased experiences of discrimination in the US during this time [56–58], however the sample of Asian American and Pacific islander youth was too small to separate out and further examine this finding using the current dataset. The post hoc marginal effects analyses for caregiver reported PSC internalizing score and youth race/ethnicity were not significant.

## Discussion

There were no differences in either caregiver or youth report of MH symptom acuity during the SAH order of the COVID-19 pandemic compared to the same time frame the previous year. This suggests the onset period of the pandemic had little or no effect on MH symptom acuity for youth seeking MH treatment.

However, we observed statistically significantly higher caregiver and youth report of MH symptom acuity at intake for youth seeking outpatient MH services in the prolonged months of the COVID-19 pandemic (after SAH order until the end of the 2020 calendar year), compared to the same time frame the previous year (2019), while controlling for youth age, gender, and sexual orientation.

The longer-term impact of the pandemic was evaluated from May to December of 2020, months 3–9 of the pandemic. Caregiver-report of PSC-35 total, attention, and internalizing scores were statistically higher in 2020 when compared with 2019, but with small effects sizes (Table 3). Caregiver PSC-35 total scores averaged 26.0 in 2019 and 27.0 in 2020. The average increase of 1.0 point (possible range of 0–70) is quite modest and both the 2019 and 2020 average scores is below the clinical cutoff score of  ≥ 28. The subscale scores were all below the clinical cutoff scores as well. In 2019 and 2020, Caregiver PSC-35 intake total scores at both time points were within the range of mean total scores reported in previous studies with outpatient pediatric psychiatry treatment-seeking populations (25.7–27.4; [46, 52, 53]).

In the prolonged pandemic cohorts, statistically higher scores were found for youth-reported PSC-35 total, attention, and internalizing scales in 2020 compared with 2019, but again with small effect sizes (Table 3). The mean youth-reported total score in 2020 was 27.9; 2.1 points higher than the 2019 mean (range 0–70; clinical cutoff score of  ≥ 28) and nearly meeting the clinical cutoff. The mean youth-reported internalizing score in 2020 was 5.2 and exceeded clinical cutoff score (≥ 5). Therefore, on average youth reported clinically significant internalizing scores in 2020 but not in 2019, and the mean youth-reported total score in 2020 exceed the intake mean total scores reported in previous clinical samples [46, 52, 53].

Youth and their caregivers reported youth were entering services experienced greater depression, anxiety, and attention problems during the prolonged period of the pandemic. The increases were significant, albeit small. However, these findings were consistent across ages (e.g., school-aged children, adolescents), gender, sexual orientation, and racial/ethnic background of the youth, indicating these findings reflect actual increases of youth mental health problems during this timeframe. This is consistent with the literature noting concerns regarding the detrimental MH impact of the pandemic (e.g., increased isolation, exclusion, and stress) and calling for prioritization of efforts to address this enduring impact on youth as a result of the pandemic [10, 22, 23].

Lastly, caregiver and youth MH symptom acuity at intake for youth seeking outpatient MH services during both phases of the pandemic were not observed to be disproportionately higher for SGD or BIPOC youth, compared to the same time frames the previous year. Although there are known baseline differences in MH symptoms among SGD and BIPOC youth populations, we found no differential response through the phases of the pandemic assessed in this study, contrary to our hypothesis. These findings diverge from the limited qualitative work by Fish and colleagues [32] noting increased MH challenges associated with the pandemic, and specifically SAH or social distancing orders. However, the literature is sparse with mixed findings for BIPOC youth. Penner et al. [35] noted the limited, and in some cases, opposite impact of the pandemic on MH among predominately Hispanic or Latinx adolescents. These results point to the continued need to examine the MH impact, especially prolonged impact, of COVID-19 among SGD and BIPOC youth to truly disentangle the contradictory findings currently in the literature. This can help inform responsive treatment efforts in coordination with the existing need to improve access of effective and appropriate MH care for SGD and BIPOC youth.

The divergent findings between the SAH and PP periods may suggest youth were experiencing resilience fatigue, a gradual exhaustion of their typical capacity to withstand stressors, as a seemingly unrelenting global pandemic demanded constant adaptation [59]. As the pandemic continued, for many it likely presented a chronic additional stressor (e.g., increased parental stress; continued separation from peers due to not having in-person school or extracurricular activities) and the cumulative effect of this stress may have led to worsening MH symptom acuity [10]. These findings are consistent with standardized reviews and meta-analysis in the area, which found that COVID-19 was related to increasing rates of depression, anxiety, and behavioral problems in youth [6–10]. Nevertheless, findings were mixed in the current study about the impact of the pandemic on behavioral problems (parents reported a decrease in behavioral problems, while youth reported no changes), both youth and their caregivers reported increased rates of anxiety and depression for youth as the pandemic prolonged [10].

### Limitations

This is among the first studies examining the prolonged impact of the COVID-19 pandemic on a large and diverse population of youth. While the data showing an increase in internalizing symptoms is clear and consistent across respondents, this study does not explain why there were increases in reported youth MH problems. This was an observational study and did not randomize youth to experience COVID-19, pandemic restrictions, to initiate MH treatment and/or COVID-19 related stressors. Increases in MH symptoms could have been due to proximal effects, such as fear of the virus, or distal factors, like school closures, not being able to access extracurricular activities, or socialize in-person. Furthermore, it is important to consider the broader societal issues also occurring during the timeframe of interest in the current work that could have affected youth in our sample. As noted in Fig. 1, significant social events transpired in 2020, including the widely viewed murder of George Floyd and subsequent global protests and an alarming rise in experiences of race-related violence toward Asian Americans and Pacific Islanders [56–58]. As such, it is impossible to disentangle the effects of the pandemic from key social justice and other nationally experienced events. Given that youth receiving community-based psychiatric services in San Diego include many BIPOC, it is reasonable to hypothesize some youth’s symptoms of depression and anxiety increased due to distress related to social injustice, but more research is needed in this area. Also, due to the limited size of our sample of youth who are SGD, it is likely there was not adequate power for our analyses to detect significant differences in symptom scores for these groups. Age cohort difference were also not examined within this study and there is currently limited research on the topic. Exploring differential responses to the pandemic based on age and developmental level could be a future area of important study. As the most common therapy modality switched from in-person therapy in 2019 to telehealth in 2020, and school referrals to MH treatment markedly slowed, symptom acuity and type of youth initiating treatment during the pandemic may have likewise shifted. However, the comparisons of demographics from the 2019 and 2020 samples of youth who received services did not uncover many differences.

## Data Availability

Not applicable.

## Abbreviations

COVID-19: Coronavirus disease 2019
PSC: Pediatric symptom checklist
MH: Mental health
SGD: Sexual and gender diverse
BIPOC: Black, indigenous, people of color
